# Doctor of Public Health-Crisis Management and COVID-19 Prevention and Control: A Case Study in China

**DOI:** 10.3389/fpubh.2022.814632

**Published:** 2022-02-04

**Authors:** Weiqin Cai, Runguo Gao, Qi Jing, Chunping Wang, Ningning Hou, Weide Liu, Qianqian Gao, Xiaodong Sun

**Affiliations:** ^1^School of Management, Weifang Medical University, Weifang, China; ^2^School of Graduate, Weifang Medical University, Weifang, China; ^3^School of Public Health, Weifang Medical University, Weifang, China; ^4^Department of Endocrinology and Metabolism, Affiliated Hospital of Weifang Medical University, Weifang, China; ^5^Clinical Research Center, Affiliated Hospital of Weifang Medical University, Weifang, China; ^6^Department of Teaching and Research, Affiliated Hospital of Weifang Medical University, Weifang, China

**Keywords:** COVID-19, public health crisis, doctor education, curriculum, scientific research

## Abstract

In the fields of public health policy and public health care, advanced educational programs are an important strategy in dealing with public health crises. The COVID-19 pandemic has exposed the global need for skilled public health leaders and managers to address complex public health challenges, which requires the strengthening of public health education at the highest levels. This paper is a qualitative case study of a special educational program for doctors of public health in China. The program's educational objectives are in line with epidemic prevention and control. With the goal of developing the world's leading national public health management system, the Chinese government established an advanced academic program for public health crisis management. The program offers doctoral students a multidisciplinary degree based upon the theoretical knowledge of crisis management, supported by advanced training in the foundational concepts, theories, and practices of public health, and the study of basic medicine which provides the theoretical support for developing essential clinical skills. Program graduates develop the theoretical, practical, and leadership-related capabilities required for the management of national emergencies. The program introduced in this paper meets current epidemic prevention and control needs and should be considered by public health policy makers, leaders, and scholars in the discussion of advanced public health policy and health care education in China, including the development of an internationally recognized Doctor of Public Health program.

## Introduction

The recent coronavirus disease (COVID-19) pandemic has exposed the global shortage of workers in the fields of public health and health care (1) and, in particular, the need for skilled leaders and managers to translate public health research into effective policies and programs to manage emerging public health threats, staff response efforts, and improve population health (2–5). To respond to the ongoing exigencies of public health and health care that have been exacerbated by COVID-19, countries have developed various emergency strategies to strengthen their public healthcare systems. For example, to support its COVID-19 contact tracing operations, Germany developed the Contact Scout Initiative to increase the nation's public health workforce with short-term labor solutions (6), and Italy mobilized its residents to create a non-professional healthcare workforce to support its national COVID-19 prevention and control measures (7). However, national public health policy makers, leaders, and scholars worldwide have recognized that these temporary strategies do not address the fundamental global public healthcare workforce crisis—and specifically, the need for skilled public health and health care leaders and managers to address complex public health challenges—that require the strengthening of public health education at the highest levels (8).

While China's public health system has made remarkable progress in proactively responding to COVID-19, it has also exposed many problems. From severe acute respiratory syndrome epidemic in 2003 to COVID-19 in 2020, the shortcomings of China's public health system have not been fully addressed. The entire public health system lags far behind in terms of personnel, technology and equipment. The growth of government's public health investment lags behind the speed of social and economic growth, and the phenomenon that “the god of wealth follows the god of misery” remains unchanged. Public health policy makers and leaders in the People's Republic of China (China) have recognized the importance of high-level leadership in the fields of public health and health care, and the need for advanced education in public health to address complex problems of public health policy in the COVID-19 era. At the national level, the Chinese government has proposed the establishment of academic institutions that provide advanced education in the foundations of public health along with the mastery of essential skills, such as pathogen identification, epidemic situation analysis and transmission law research, and field epidemiological investigation (9). Thus, the Chinese government has acknowledged the fundamental role of advanced public health education in the vigorous cultivation of future leaders who will manage public health issues and play an essential role in preventing and controlling infectious diseases (10), translating academic knowledge and skills into practical results in the field.

The cultivation of advanced scholarship combined with leadership and applied practice skills among healthcare professionals has become an essential goal in public health policy development (11). The doctorate in public health represents the most advanced level of formal education available in the field (12). Graduate students in public health at the doctoral level include the Science degree doctoral education (Ph.D.) and the professional degree (DrPH). Ph.D. focuses on training research-oriented talents in a certain field of public health to solve scientific problems arising from basic public health research, while DrPH focuses on training leaders in the field of public health to solve practical problems arising from public health (13). Among them, the DrPH graduate, who has received a comprehensive multidisciplinary education encompassing academic, applied public health, and leadership training, has been prepared to lead transformational organizational and societal efforts in public health and health care (14).

However, at the time of publishing, China had not yet established a national DrPH program, although Chinese universities are exploring various DrPH programs (11). For example, in 2020, Xi'an Jiaotong University, Peking University, Fudan University, Zhejiang University, and other universities jointly proposed the establishment of a Chinese DrPH degree program (15). In this paper, we focus on the case study of a unique advanced academic degree in public health and crisis management offered by a Chinese medical school that was developed to cultivate knowledge and skills at the doctoral level in the practice of public health with a special focus on crisis management. Graduates of this program have been among the leaders of the prevention and control measures in the COVID-19 pandemic in China. This case study has implications for scholars and public health policy makers in the development of the similar advanced multidisciplinary academic programs in the fields of public health and health care.

## Materials and Methods

### Setting

In 2011, the Academic Degrees Committee of China's State Council issued the Opinions on the Pilot Program of Personnel Education to Serve the Special Needs of the State. According to these opinions—which outlined China's urgent need for scholarly and practical excellence in public health policy and health care—the law provided for a pilot doctoral degree program at several advanced academic institutions in China, called the Talent Education Program for National Special Needs, with doctoral degree granting. Although this was not a DrPH program, the State Council granted Weifang Medical University, Weifang, Shandong Province, permission to offer a public health curriculum with a focus on crisis management—the Doctoral Training Program of Public Health-Crisis Management (DrTP-PHCM). The recruitment of full-time doctoral students began in 2013. At the time of publishing, 54 students have been enrolled in the University's DrTP-PHCM, including 16 graduates and 38 students currently attending the school.

### Methods

The data sources of this study mainly include: (1) The system documents of DRTP-PHCM are sorted out, mainly including the education plan and curriculum setting. From the education plan, the education objectives of the program are sorted out, and the main curriculum setting is introduced. (2) The basic information of Ph.D. students recruited by DRTP-PHCM since its establishment is collected, mainly their scientific research, internship and international exchange, and graduation destination are sorted out and analyzed.

## Results

### Education Objectives

**Figure 1:**
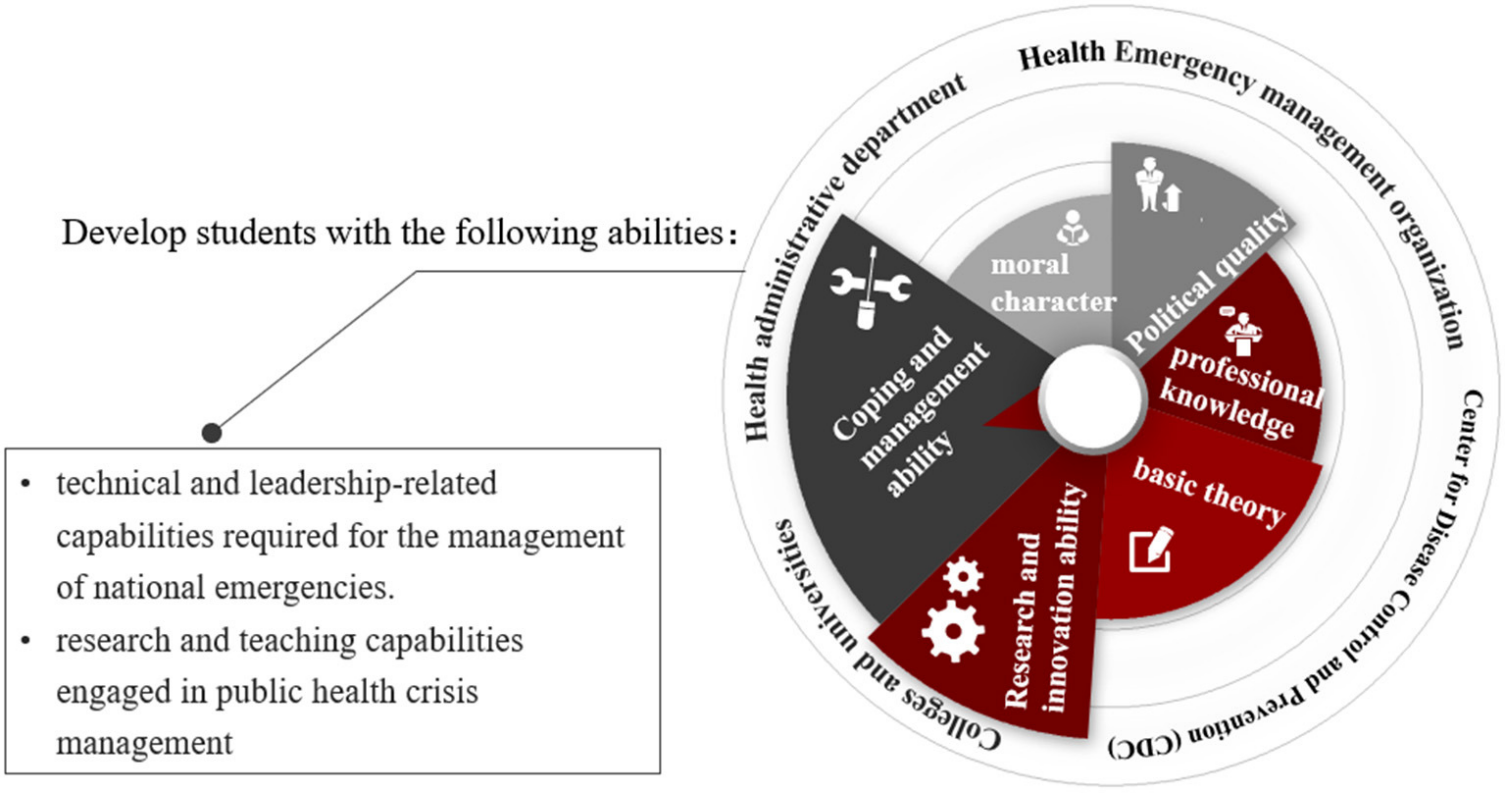
Education objectives of DrTP-PHCM.

Figure 1 shows the education objectives of DrTP-PHCM. The DrTP-PHCM of Weifang Medical University offers a multidisciplinary degree based upon the theoretical knowledge of crisis management, supported by advanced training in the foundational concepts, theories, and practices of public health, and the study of management and psychology which provides the theoretical support for developing the cultivation of comprehensive professional skills of public health crisis management. As shown in Figure 1, DrTP-PHCM graduates develop the technical and leadership-related capabilities required for the management of national emergencies, as well as the research and teaching skills needed to engage in public health crisis management. The students can serve not only in universities and scientific research institutions, but also in health administration departments, health crisis management departments and disease prevention and control institutions.

The educational objectives of the DrTP-PHCM are consistent with China's national public health security needs, and the Program offers students the opportunity to deepen areas of expertise and build skills in three specific areas—public health crisis management theory and policy research, public health crisis prevention and response, and public health crisis psychological intervention. Since the establishment of the Program, up to 5 students have been enrolled in each study area annually.

### Curriculum

To achieve the objectives of China's Ministry of Education, the DrTP-PHCM developed a curriculum that meets specific educational requirements. Doctoral students are required to take a minimum of 24 credits during the full program. The Program has constructed a curriculum integrating public health and crisis management foundation courses, professional core courses, hands-on experiential courses, and courses designed to promote innovative thinking (Table 1). Professional foundation courses and professional core courses cover public health policy, public health and crisis management, and other related educational objectives. The experiential courses include simulation exercises, applied practice, and case studies. The courses to develop innovative thinking include lecture participation, scientific research training, and studying abroad. Regarding student assessment, public courses, and professional foundation courses and elective courses generally require comprehensive examinations, experts evaluate hands-on practical training, and a dissertation is part of the graduation requirements.

**Table 1:** Curriculum system of DrTP-PHCM.

**Category**	**Course**	**Assessment method**
Public Courses	Marxism in Chinese, English Reinforcement, English Thesis Writing, etc.	Examination
Professional Foundation Courses	Read Some Classic Works of Public Health Management, Sociological Research Methods, Health Security System and Reform, etc.	Examination
Professional Core Courses	Public Health Policy, Overview of Public Health Crisis, Early Warning and Crisis Management, Methods and Techniques of Public Health Field Investigation, Social Psychology of Disaster Response from an International Perspective, etc.	Examination
Experiment and Practice Course	Innovative Experiment, Simulation Exercise and Base Practice, Case Study, etc.	Diversified Test
Innovative Course	Frontier Lectures and Seminars, Scientific Research Training, Academic Exchange, Studied Abroad, Subject Implementation, Thesis Writing, etc.	Diversified Test

### Scientific Research

Since the establishment of the DrTP-PHCM of Weifang Medical University, teams of doctoral advisors and candidates have conducted scientific research about public health and crisis management. In their role as doctoral advisors, members of the University faculty have cooperated with the Chinese Center for Disease Control and Prevention, the Chinese Health Development Research Center, and other institutions. To address major societal risks in the fields of public health and health care, these teams assessed public health vulnerability, crisis early warning mechanisms, individuals' psychological status, and emergency strategies and countermeasures that deal with public health crises.

The DrTP-PHCM degree requires that graduate students' dissertations be oriented to meet China's national public health and crisis management needs, and thus can directly support government decision-making, or guide epidemic prevention and control strategies. Since the establishment of the Program, graduate students' dissertations have focused on the three aforementioned study areas—public health and crisis management theory and policy, public health crisis prevention and response, and public health crisis psychological intervention—and as of September 2021, 16 doctoral candidates had completed their dissertations (Table 2).

**Table 2:** Scientific research of DrTP-PHCM.

**Specific areas (number of dissertations)**	**Dissertation topic**	**Research direction**	**Focus problem**
Public Health Crisis Management Theory and Policy (5 dissertations)	Public health security strategy of China	Public health Security	National public health security
	Research on the risk early-warning of China total expenditure on health	Public health burden	Risk identification of total health expenditure
	Research on risk of government health expenditure in China	Government public health expenditure	Risks of government health expenditure
	Research on the Relationship between Health Input and Health Output in OECD Countries from the Perspective of Public Risk	Government public health input	Relationship between health input and health output
	Research on the competency model of public health rapid response team and intervention based on emergency exercise	Public health leadership	National health emergency manpower competence
Public Health Crisis Prevention and Response (6 dissertations)	Research on vulnerability of village clinic doctors in Shandong province in the context of the new medical reform	Primary public health workforce	Vulnerability of rural doctors
	Research on emergency management capability of hospital infection outbreak in third-level general hospital	Public health emergency management of tertiary general hospital	Emergency response capacity of nosocomial outbreak in Tertiary General Hospital
	Development and empirical study of leadership evaluation tools for infectious disease prevention and control managers	Public health leaders	Leadership evaluation of infectious disease prevention and control managers
	Research on the development characteristics and countermeasures of internet public opinion on public health emergencies	Response to public health emergencies	Network public opinion on public health emergencies
	Research on evaluation and optimization strategy of Shanghai infectious disease prevention system	Public health emergency management	Evaluation of infectious disease prevention and control system
	Research on community resilience evaluation and adaptive Governance of the emergence of major infectious diseases	Community resilience to sudden major infectious diseases	Community resilience evaluation and adaptive management of sudden infectious diseases
Public Health Crisis Psychological Intervention (4 dissertations)	Research on psychology crisis vulnerability of community residents	Psychological crisis intervention in public health emergencies	Psychological crisis of community residents
	The genetic basis of impulsivity and the neuroelectrophysiological mechanism of inhibitory function in depression patients with suicidal ideation and intervention strategies	Psychological crisis intervention in public health emergencies	Suicide ideation in patients with depression
	Cognitive reappraisal intervention for anxiety and depression in breast cancer survivors with post-traumatic stress symptoms	Psychological crisis intervention in public health emergencies	Post-traumatic stress symptoms, anxiety, and depression in breast cancer survivors
	The effect of forgiveness and social connectedness in adolescents' depression severity on suicidal ideation: a mechanism and intervention study	Psychological crisis intervention in public health emergencies	Adolescent depression and suicidal ideation

### Hands-On Training

The University's DrTP-PHCM allows doctoral candidates to select a personalized training programs based on individual interests and qualifications. In the Program, doctoral students are required to have at least 6 months of overseas learning experiences, international academic exchanges, or professional practice experiences under the guidance of a faculty member. At present, except for first-year students studying professional foundation courses, students have completed or are participating in hands-on training to develop their professional practical skills.

The Program requires doctoral students complete practical training in health emergency response institutions, disease prevention and control institutions, and other practice institutions for at least 6 months. The University has established joint doctoral training bases with the Chinese Center for Disease Control and Prevention (CDC), the Health Emergency Response Office of the National Health Commission, and the Research Center for Health Development of the National Health Commission. The University cooperates with the Health Commission in Shandong Province, Fudan University, and Harbin Medical University to jointly train students and has hired part-time tutors and practical training instructors for the foundational programs. In addition, the University's students can participate in exchange programs with the University of South Wales or can apply to other foreign universities with programs related to their majors. Table 3 shows the practical training of doctoral students. At present, all current and previous students have participated in internship training; however, due to the impact of COVID-19, few students have participated in overseas studies or international academic exchanges.

**Table 3:** Hands-on training of DrTP-PHCM.

**Practical Form**	**Institutions**	**Practice contents**
Professional Practice Experience	CDC, Health Emergency Response Office of the National Health Commission, China Armed Police General Hospital, National Health Development Research Center of the National Health Commission, Center for Health Development Strategy of Fudan University, etc.	Learn the techniques of public health crisis monitoring, early warning, disposal, protection and management
Overseas Learning Experience	Manchester Metropolitan University, UK	Learn about the current system of PHCM in the UK and learned from the experience of foreign PHCM
	Ebendorf Medical Center, University of Hamburg, Germany	Research on public health issues such as adolescent bullying
	World Health Organization Western Pacific Region	Participate in the risk assessment of new public health emergencies
	University of South Wales, UK	Learn about PHCM theory
International Academic Exchange	Yale University	Presented conference report entitled regional comparative study of medical resource allocation efficiency in various community health service stations in Shandong Province
	Health Technology Assessment International Conference	Study on process of China's Essential Medicine System based on Smith-Model
Social Service	Chinese CDC	Psychological intervention of typhoon “Lekima” flood disaster (2018)
	Chinese CDC	Collation of domestic and foreign information, data analysis and report writing. Handle other urgent tasks (2020)
	Shandong CDC	Participated in volunteer services for COVID-19 prevention and control in Shandong province (2020)
	Gansu Working Group of The State Council Joint Prevention and Control Mechanism	Epidemiological survey of public health crisis (2021)

In addition, doctoral students practice and develop skills while engaging in hands-on projects that allow them to actively contribute to public health, healthcare, or crisis situations. For example, in August 2018, flood disasters occurred in Shouguang and Qingzhou in China's Shandong Province. In a widely praised initiative, the University's DrTP-PHCM teams organized psychological crisis intervention experts and doctoral students conducted post-disaster psychological assistance in the disaster areas. On January 19, 2020, eight DrTP-PHCM doctoral students participated in a COVID-19 epidemiological group investigation and other related work of the Chinese CDC, which subsequently sent a letter of commendation praising the doctoral students for their COVID-19 prevention and control activities. On October 19, 2021, the severe COVID-19 situation in China's Gansu Province led the National Health Commission to select Weifang Medical University faculty members and DrTP-PHCM doctoral students to join the State Council Joint Prevention and Control Gansu Working Group to conduct an investigation of the COVID-19 epidemic in the Province. Through hands-on practical experience, doctoral students can master the crisis management technologies of monitoring, early warning, disposal, and protection, and meet the national demand for high-level public health crisis management. The specific practices are shown in Table 3.

### Graduate Career Paths

**Figure 2:**
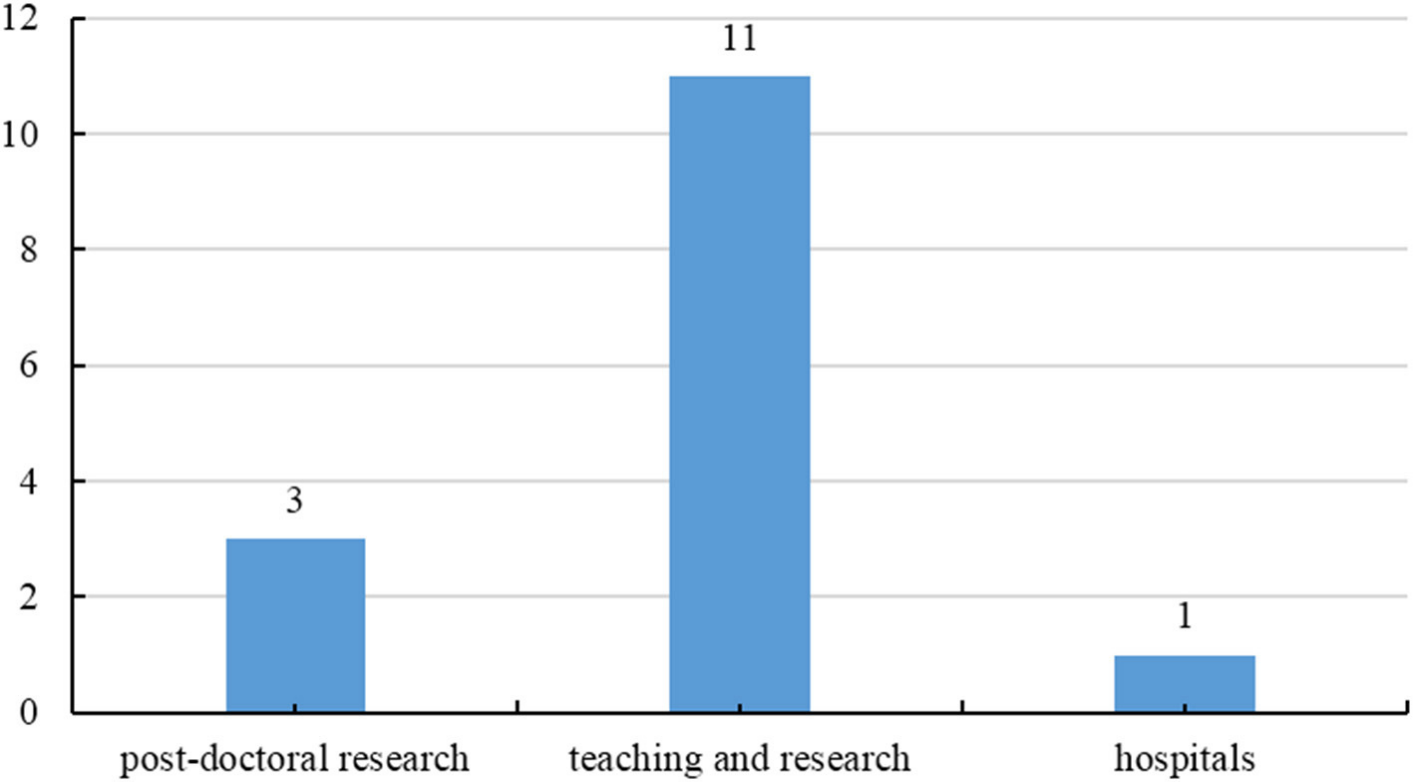
Graduate career paths of DrTP-PHCM.

Since the inception of the DrTP-PHCM in 2013, 15 doctoral students have been employed in public health crisis management. Figure 2 shows graduates' career paths. Three are engaged in emergency management research at post-doctoral research facilities of well-known universities in China, 11 are engaged in teaching and research about public health and crisis management in universities, and one is engaged in public health management in hospitals. The graduates' professional abilities, research competencies, and leadership qualities have been well-received by leaders and colleagues in the fields of public health policy, public health care, and crisis management.

## Discussion

The development of an advanced public health workforce is a powerful weapon in dealing with national emergencies and major public health crises (16), and the DrTP-PHCM of Weifang Medical University is well-positioned for the response and management needs of major public health crises, including the current prevention and control measures of the COVID-19 pandemic. The Program's graduate students have demonstrated that they can play an important role in the response to and management of public health emergencies—eight Weifang Medical University students working on the COVID-19 epidemiology team at the Chinese CDC have received a letter of commendation from the CDC, and the objectives of the program have been widely recognized by China's National Public Health Department and Society, validating the vision and execution of the Program.

The consensus is that the training of public health manpower should attach importance to practical application (5, 17). Public health and health care policy-makers and leaders worldwide attach great importance to the development of doctors' practical abilities. For example, the School of Public Health of the University of Kentucky requires that every student complete 9 weeks of education in one or more public health places (18), and has established a positive relationship with these institutions, providing doctoral students the opportunity to learn advanced health emergency competencies, including epidemic situation analysis and the study of the regular transmission pattern of infectious diseases, field epidemiology investigation, epidemic data collection and analysis, practical ability, practical ability of health emergency and rescue. At the same time, applied practice provides an important, hands-on opportunity for students to implement classroom learning of public health knowledge and skills in a real-world setting, such as participating in front-line COVID-19 pandemic prevention and control activities.

With the frequent occurrence of public health emergencies, the population's demand for public health services has been increasing, and public health policy and health care professionals have had to perform at increasingly higher levels (19). DrPH graduate students have contributed to improving public health policy and health care by applying knowledge and skills in executive leadership positions; measuring, monitoring, and promoting the operation and performance of international health service organizations; promoting social and organizational change; and becoming outstanding public health leaders (13). Many countries in the world have established a perfect DrPH training mode while establishing academic doctoral training. For example, the United States was the first country to award a DrPH, when Harvard University awarded a DrPH in 1911 (8). Some universities in China have experimented with DrPH training. Xi ‘an Jiaotong University and Peking University started DrPH cultivation pilot programs in 2015 and 2016, respectively. According to the feedback of the graduates, they believe that their practical ability and interpersonal communication ability have been significantly improved during the training process, and their ability to actively discover, analyze and solve public health problems has been improved through social practice (15).

The initiatives of Weifang Medical University in conducting doctoral education in public health and crisis management has been of significance to policy makers, leaders, and scholars in the fields of public health and health care (20). At time of publication, there is no DrPH program in China, so the DrTP-PHCM can only educate doctoral students and provide academic degrees—which is the biggest limitation of the Weifang Medical University program. So the result is that most graduate students of DrTP-PHCM work in research institutions and colleges or continue their studies in institutions. Few are in executive management positions in national or local disease control centers or health departments. If a DrPH education plan can carry it out, the program's education mode and various settings may be more orderly. Chinese scholars have repeatedly proposed that China establish a professional doctoral education program in public health policy and health care (15, 21).

This study had a few limitations. First, this study only introduces the DrTP-PHCM of Weifang Medical University as a case, and does not compare with similar programs. As mentioned in the previous materials and methods section, the program was set up with a special national policy background. At the same time, public health crisis management is a non-traditional public health major, but was set up to meet the major social needs of public health crisis response and management in China. Second, the program has only been in operation for 8 years from 2013 to now. During this period, the experience and lessons accumulated by the project are co-existing. Therefore, this study hopes to absorb more suggestions and opinions through the exhibition of the program to help the development of doctor education of public health in China.

## Conclusions

The advanced education of highly qualified public health policy and health care professionals is critical to effectively dealing with major global public health crises. The DrTP-PHCM—the public health crisis management program of Weifang Medical University introduced in this paper—meets current epidemic prevention and control needs and should be considered by public health policy makers, leaders, and scholars in the discussion of advanced public health policy and health care education in China, including the development of an internationally recognized Doctor of Public Health program.

## Data Availability Statement

The raw data supporting the conclusions of this article will be made available by the authors, without undue reservation.

## Author Contributions

WC and QG conceived the idea, investigated and analyzed the data, and wrote the initial draft of the paper. RG, QJ, CW, NH, and WL participated in discussion and revision in this study. XS conceived the idea and revised the manuscript. All authors contributed to the final manuscript and agreed on the final version.

## Funding

This study was funded by the National Natural Science Foundation of China (grants 72104186, 81870593), Humanities and Social Science Research Youth Fund program of Ministry of Education (grants 20YJCZH002), Natural Science Foundation program of Shandong Province (grants ZR202102180607, ZR2020MH106), the Quality Improvement of Postgraduate Education in Shandong Province (grants SDYAL19156), and Research Project of China Academic Degree and Graduate Education Society (B3-YX20180305-04).

## Conflict of Interest

The authors declare that the research was conducted in the absence of any commercial or financial relationships that could be construed as a potential conflict of interest.

## Publisher's Note

All claims expressed in this article are solely those of the authors and do not necessarily represent those of their affiliated organizations, or those of the publisher, the editors and the reviewers. Any product that may be evaluated in this article, or claim that may be made by its manufacturer, is not guaranteed or endorsed by the publisher.
